# Double-seronegative neuromyelitis optica: it is possible to interrupt treatment after 10 years of stability *

**DOI:** 10.1055/s-0045-1814371

**Published:** 2026-02-04

**Authors:** Guilherme Diogo Silva

**Affiliations:** 1 Universidade de São Paulo, Faculdade de Medicina, Hospital das Clínicas, Divisão de Neurologia, Grupo de Neuroimunologia, São Paulo SP, Brazil.

**Keywords:** Neuromyelitis Optica, Aquaporin 4, Myelin-Oligodendrocyte Glycoprotein, Immunotherapy, Therapeutics

## Abstract

Double-seronegative neuromyelitis optica spectrum disorder (DS-NMOSD) encompasses a heterogeneous spectrum, including monophasic and relapsing phenotypes. While 1/3 patients may follow a monophasic course, lifelong immunotherapy remains common practice due to the fear of relapse. However, this strategy may unnecessarily expose stable patients to long-term adverse effects and economic burden. The condition lacks the relapse-associated mechanisms observed in aquaporin 4 (AQP4)-positive disease, questioning the generalizability of prior treatment-withdrawal studies. Although predictive markers for relapse are still lacking, the median time to relapse is of approximately 3.4 (range: 0–7) years; hence, sustained remission beyond 10 years may indicate a subgroup of patients with low relapse risk. Until prospective data are available, individualized, cautious treatment interruption should be considered, guided by shared decision-making.

Double-seronegative neuromyelitis optica spectrum disorder (DS-NMOSD) represents a heterogeneous clinical syndrome rather than a single disease entity. It encompasses a range of presentations, from monophasic, postinfectious illnesses to relapsing courses that are clinically indistinguishable from NMOSD positive for aquaporin 4-immunoglobulin G (AQP4-IgG). 1 This heterogeneity complicates treatment decisions—especially the question of whether long-term immunotherapy can be safely discontinued in patients with a prolonged period of clinical stability.

A systematic review of DS-NMOSD 2 found that approximately 1/3 patients follow a monophasic disease course. This proportion is substantial, suggesting that a meaningful subset of patients may never experience a relapse; therefore, they may not benefit from indefinite immunosuppression. Moreover, a ‘benign’ NMOSD phenotype (score ≤ 3.0 on the Expanded Disability Status Scale [EDSS] at ≥ 15 years) was described in 3 to 5% of the patients, which could suggest the possibility of treatment discontinuation in selected cases. 3 4 Continuing therapy in such patients not only exposes them to potential adverse effects—including infections, malignancies, and drug toxicity—but also imposes long-term economic and psychological burdens. Mortality analyses in AQP4-IgG-positive NMOSD report that approximately 10% of deaths were attributable to infection or cancer, underscoring the safety trade-off of indefinite therapy. This is particularly relevant for pediatric patients, who start immunosuppression very early, and cases reports 5 6 have described prolonged remission following cessation of rituximab treatment. Although we can use strategies to reduce this burden—such as vaccinations 7 8 9 and rational selection of immunosuppressants following cost-effective models 10 —there is a compelling clinical and ethical rationale to reconsider the necessity of lifelong treatment in DS-NMOSD patients who have remained relapse-free for many years, particularly in countries with a high prevalence of NMOSD, such as Brazil. 11 However, the critical challenge lies in identifying which patients truly belong to this monophasic group. To date, no biomarker or clinical feature has proven sufficiently reliable to confidently differentiate monophasic from relapsing DS-NMOSD. Several factors have been associated with relapse risk. Later age at onset has emerged as a consistent predictor of recurrence in DS-NMOSD. This association was first reported in an observational study 12 and later confirmed in a meta-analysis. 2 Conversely, certain features have been proposed as protective, including the use of immunosuppressive therapies—especially rituximab 13 —and the simultaneous occurrence of optic neuritis and transverse myelitis. 12 Yet, despite these associations, none has demonstrated sufficient predictive value to confidently guide individualized treatment-cessation decisions.

Understanding the natural history of relapse in DS-NMOSD further informs the discussion. Among patients who relapse, the mean interval from the first to the second attack is of 3.4 years, with a wide range, from 0 to 7 years. 14 This pattern suggests that patients who remain stable for a full decade may, in fact, represent a biologically-distinct subgroup with low relapse risk. The passage of such a prolonged relapse-free period without treatment failure raises the legitimate question of whether continuing therapy offers any incremental benefit in these cases.

Despite this rationale, much of the hesitation around stopping treatment in NMOSD stems from studies focused on AQP4-IgG-positive patients. In a well-known case series, 15 17 AQP4-IgG-positive NMOSD patients discontinued immunotherapy after a median relapse-free interval of 62 (interquartile range [IQR]: 52–73) months. Alarmingly, 82% of them relapsed within a median of 6 (IQR 4–34) months. 15 This high relapse rate strongly supports continued therapy in AQP4-IgG-positive NMOSD, even after long periods of clinical stability. However, extrapolating data from AQP4-IgG patients to DS-NMOSD might be misleading for clinicians. The distinct pathophysiology has an impact on relapse probability and the safety of therapy discontinuation. Notably, 29% of patients in this cohort 15 were seronegative at the time of treatment discontinuation, but 80% of them showed seroconversion at the time of relapse—suggesting a pathophysiological mechanism driven by the re-emergence of anti-AQP4 antibodies. Since DS-NMOSD patients, by definition, lack AQP4-IgG, they are unlikely to share this specific relapse mechanism, further supporting the notion that the risks of treatment interruption may not be equivalent between seropositive and double-seronegative cases.

Additional insight can be drawn from studies on myelin oligodendrocyte glycoprotein-immunoglobulin G (MOG) antibody-associated disease (MOGAD), a condition that overlaps clinically with NMOSD but has a distinct immunopathogenesis. In MOGAD, the persistence of anti-MOG IgG is a strong predictor of relapse, 16 and its disappearance is often used as a criterion to justify avoiding long-term immunotherapy, as many patients present a monophasic course. Some cases of DS-NMOSD might occur from misclassification of AQP4-IgG- or MOG-IgG-positive patients who received false-negative results due to the influence of assay timing and technical limitations. Many studies 17 on MOG status in DS-NMOSD patients did so in the chronic phase, despite evidence that antibody titers decline over time. Approximately 25% of MOGAD patients test low-positive by 6 months, and up to 50% test negative at 12 months following a clinical attack. These findings reinforce the idea that antibody testing—when performed outside the optimal diagnostic window—can lead to false-negative classifications and may explain the apparent ‘0double seronegativity’ in some patients who undergo a monophasic disease course. Repeat testing for AQP4-IgG and MOG-IgG should be considered in patients with high clinical suspicion if live cell-based assays were not performed or if only tested in the chronic phase of disease after immunotherapy.

Further support for reconsidering continuous therapy in DS-NMOSD comes from analogous data in other double-seronegative central nervous system demyelinating syndromes. In a case series 18 of patients with relapsing idiopathic myelitis who tested negative for AQP4 and MOG antibodies, all 11 individuals who had remained relapse-free for more than 5 years safely discontinued treatment, with no relapses observed over a median follow-up of 36.5 months. While small, this study 18 adds to the accumulating body of evidence suggesting that treatment interruption may be safe in selected double-seronegative cases.

Patient-centered decisions should be based on the balance between the risk of relapse-associated disability against the adverse effects of long-term immunosuppression. A practical recommendation is to consider discontinuing immunotherapy only if all four of the following criteria are met:

- ≥ 10 years of sustained clinical remission;
- low and stable disability on serial assessments (persistently low EDSS scores);
- repeatedly negative high-sensitivity live cell-based assays for AQP4-IgG and MOG-IgG; and
- informed, shared decision-making with a predefined monitoring and relapse-response plan.

However, the current knowledge on immunotherapy discontinuation in DS-NMOSD is based on retrospective case series and observational data. High-quality, prospective, multicenter studies are lacking, and current evidence remains preliminary. There is a pressing need for prospective validated biomarkers and risk stratification tools to guide personalized safe de-escalation strategies. The problem of treatment discontinuation in DS-NMOSD reinforces the need for well-designed, multicenter prospective trials and biomarker development to establish safe, evidence-based de-escalation and drug discontinuation protocols.
